# Molecular-replacement phasing using predicted protein structures from *AWSEM-Suite*

**DOI:** 10.1107/S2052252520013494

**Published:** 2020-10-27

**Authors:** Shikai Jin, Mitchell D. Miller, Mingchen Chen, Nicholas P. Schafer, Xingcheng Lin, Xun Chen, George N. Phillips, Peter G. Wolynes

**Affiliations:** a Center for Theoretical Biological Physics, Rice University, Houston, Texas, USA; b Department of Biosciences, Rice University, Houston, Texas, USA; c Department of Chemistry, Rice University, Houston, Texas, USA; d Department of Chemistry, Massachusetts Institute of Technology, Cambridge, Massachusetts, USA; e Department of Physics, Rice University, Houston, Texas, USA

**Keywords:** *AWSEM-Suite*, molecular replacement, structure prediction

## Abstract

The phase problem in X-ray crystallography arises from the fact that only the intensities, and not the phases, of the diffracting electromagnetic waves are measured directly. Molecular replacement can often estimate the relative phases of reflections starting with those derived from a template structure, which is usually a previously solved structure of a similar protein. The key factor in the success of molecular replacement is finding a good template structure. When no good solved template exists, predicted structures based partially on templates can sometimes be used to generate models for molecular replacement, thereby extending the lower bound of structural and sequence similarity required for successful structure determination. Here, the effectiveness is examined of structures predicted by a state-of-the-art prediction algorithm, the *Associative memory, Water-mediated, Structure and Energy Model Suite* (*AWSEM-Suite*), which has been shown to perform well in predicting protein structures in CASP13 when there is no significant sequence similarity to a solved protein or only very low sequence similarity to known templates. The performance of *AWSEM-Suite* structures in molecular replacement is discussed and the results show that *AWSEM-Suite* performs well in providing useful phase information, often performing better than *I-TASSER-MR* and the previous algorithm *AWSEM-Template*.

## Abstract 2

This paper describes and evaluates the performance of *AWSEM-Suite*, an algorithm that includes template-guided refinement and coevolutionary information within the framework of the energy-landscape theory, in using molecular replacement to solve the phase problem by the *de novo* prediction of structures. It also highlights and discusses how *AWSEM-Suite* provides better predictions than *I-TASSER-MR* or the previous algorithm *AWSEM-Template*.

## 1. Introduction   

For more than 60 years, the rationalization of protein crystallogenesis and the finding of readily usable solutions for the phase problem have been important technical challenges for protein crystallographers (Abergel, 2013 ▸). In X-ray crystallo­graphy, the crystallographer seeks to obtain an electron-density map from the diffraction pattern produced by X-rays. The X-ray diffraction experiment, however, only records the intensities of the diffracted waves and not their relative phases. The so-called ‘phase problem’ thus results since such phases are not measured directly but are still required for the calculation of the electron-density map. Molecular replacement is an approach that does not require any further experiments such as isomorphous replacement or anomalous dispersion to estimate the necessary phase information. Molecular replacement, therefore, is often the most economical and the fastest method for solving the phase problem in X-ray structure determination. Molecular replacement accounts for around 80% of the molecular structures deposited in the Protein Data Bank (PDB) to 2017 (Wang, Virtanen *et al.*, 2017 ▸).

Presently, molecular replacement requires the accurate knowledge of the structure of a homologous protein that is similar enough to the target to serve as a template so that one can find the correct placement of the template structure in the unit cell of the unknown protein. Although there are exceptions, the root-mean-squared distance (r.m.s.d.) between the atomic positions of the template model and the final structure usually needs to be less than 2.0 Å (Abergel, 2013 ▸; Scapin, 2013 ▸). The success rate of molecular replacement decreases rapidly as sequence identity falls below 30% (Scapin, 2013 ▸). Unfortunately, for roughly 41% of protein families there is currently no member with known structure (Kamisetty *et al.*, 2013 ▸). With the development of proteomics in recent years, increasing absolute numbers of protein sequences with no known structural homologs have emerged. Thus, using predicted structural models as templates in molecular replace­ment has much potential.

*Ab initio* protein structure prediction with high fidelity and resolution remains a challenge in computational biology (Croll *et al.*, 2019 ▸). Nevertheless, the CASP experiments held every two years have witnessed a fairly steady improvement in the backbone accuracy of the best models. Structure-prediction methods now include deep-learning-based prediction, molecular-dynamics simulation and a variety of schemes of homology building from templates. Several protocols, including *MR-Rosetta*, *QUARK* and *I-TASSER-MR*, that have participated in the CASP experiments have been used to solve the phase problem (Qian *et al.*, 2007 ▸; Keegan *et al.*, 2015 ▸; Wang *et al.*, 2016 ▸).

One prediction algorithm, the *Associative memory, Water-mediated, Structure and Energy Model* (*AWSEM*), is based on the principles of energy-landscape theory and protein-folding funnel theory (Bryngelson *et al.*, 1995 ▸; Davtyan *et al.*, 2012 ▸). *AWSEM* has shown success in structure prediction for both monomers and dimers, and has been used to understand protein aggregation and the dynamics of larger assemblies, even those including DNA (Chen *et al.*, 2016 ▸, 2017 ▸; Zheng *et al.*, 2012 ▸). In this paper, we use the latest version of *AWSEM*, *AWSEM-Suite*, with a realistic coarse-grained force field that combines any homologous tertiary-structure information and coevolutionary information into a physics-based algorithm containing transferable energy terms to construct template structures, to solve the phase problem (Jin, Chen *et al.*, 2020 ▸). Since *AWSEM-Suite* is a coarse-grained molecular-dynamics model, running it is computationally faster than any of the abovementioned protocols, especially for large proteins. To explore the capability of the algorithm to make phase predictions where there are only low-quality templates, in this paper we focus on making predictions for distantly related protein targets, where the protein structures available have less than 30% sequence identity.

In this study, we evaluated the overall performance of *AWSEM-Suite* and compared its performance with those of *I-TASSER-MR* and the previous code *AWSEM-Template*. *AWSEM-Suite* provides better models for molecular replacement than those used previously. We also evaluated the quality of each predicted structure in several ways and correlated the statistics of various quality metrics with the success rate in phasing. Because *AWSEM-Suite* relies on statistical sampling, we also analyzed whether additional simulations to increase the amount of sampling would lead to greater success in blind prediction in those cases where the standard protocol initially failed to find a correct molecular-replacement solution.

## 2. Methods   

### 2.1. The *AWSEM-Suite* prediction algorithm   

*AWSEM* is a coarse-grained force field that relies on the software framework of the *LAMMPS* open-source software package to carry out simulations (Plimpton, 1995 ▸). The source code and installation instructions are open and freely available for download at http://awsem-md.org/. Only three atoms per residue are made explicit in these simulations. These fiducial atoms are the C^α^, C^β^ and O atoms of each amino acid, with the exception of glycine, which lacks a C^β^ atom. The locations of the three other backbone atoms, C′, N and H, are then inferred based on assuming ideal amino-acid geometry. The detailed structure of the side chains and the accompanying solvent are not explicitly present in the model. The force field in *AWSEM-Suite* contains both optimized transferable tertiary energy terms and specific knowledge-based terms constructed using a neural network associative memory Hamiltonian (Jin, Chen *et al.*, 2020 ▸). The tertiary interactions are transferable so that one set of parameters can be used for all sequences. These parameters have been optimized by an energy-landscape theory-based learning algorithm. Readers interested in the details of this model can consult the review by Schafer *et al.* (2014 ▸). The energetic constraints within the *AWSEM-Suite* Hamiltonian are listed in (1): 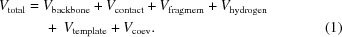

*V*_backbone_ applies a restraint through a harmonic potential to constrain the peptide backbone to an ideal geometry and to avoid overlapping of the fiducial atoms. *V*_contact_ is made up of two parts: *V*_direct_ and *V*_water_. *V*_direct_ describes effective short-range interactions between the C^β^ atoms (C^α^ for glycine) in different residues. *V*_water_ is a many-body interaction term that switches between a water-mediated and a protein-mediated interaction depending on the local density of residues around the interacting residues. The associative memory term, *V*_fragmem_, biases the formation of local secondary and supersecondary structures based on the matching of overlapping peptide fragments of known structures with the input sequence. *V*_hydrogen_ describes the formation of hydrogen bonds in α-helices and β-sheets. *V*_template_ and *V*_coev_ are newly added terms (Chen *et al.*, 2018 ▸; Sirovetz *et al.*, 2017 ▸). *V*_template_ uses a collective variable *Q*_template_ to measure the similarity to an input template structure. *Q*_template_, which ranges between 0 and 1, measures the structural similarity by comparing pairwise distances,
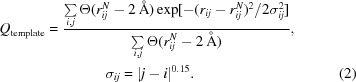

*Q*_template_ is computed using only the aligned regions of the template in the pairwise sum. Θ(*r^N^_ij_* − 2 Å) is 1 for *r^N^_ij_* ≥ 2 Å and 0 otherwise. *r^N^_ij_* is the residue–residue distance in the templates, while *r_ij_* is the pairwise distance in the simulation snapshot to which the template refers. The *V*_coev_ term stabilizes a specified choice of contacts that can be specified as input. Usually, these inferred contacts come from coevolutionary algorithms. The location of the well centers that indicates the distance constraint range for each specified contact pair depends on the identity of the paired residues. The reference distance for each possible pair of amino-acid types is based on a survey of thousands of PDB structures (Sirovetz *et al.*, 2017 ▸).

### 2.2. Detailed simulation protocol for using *AWSEM-Suite* for molecular replacement   

**Figure 1:**
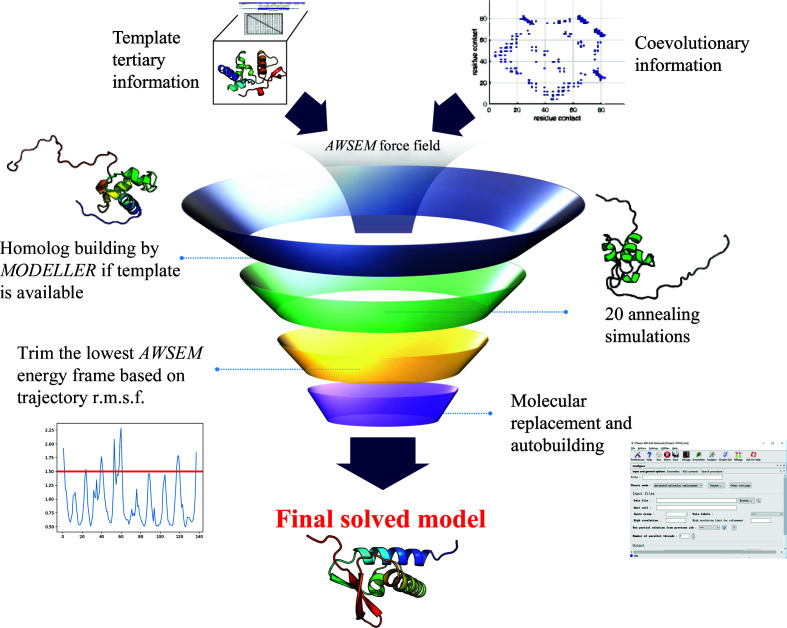
Protocol of *AWSEM-Suite* structure prediction for molecular replacement.

The simulation protocol used in *AWSEM-Suite* structure prediction is sketched in Fig. 1 ▸. In order to predict the protein structures in a blind way in this paper, we have only employed distant homologues in modeling and have always chosen a template with less than 30% sequence identity. Of course, when not performing a testing exercise, as we are here, one would normally use the best template that is available, which would increase the likelihood of phasing success. The templates were identified using *HHPred*, a hidden Markov model for sequence-database searching and multiple sequence alignment (Zimmermann *et al.*, 2018 ▸). The fragment memory term in the present implementation utilizes the local-in-sequence structure information from known experimental structures. For these test simulations, we only used those sequences with an overall sequence identity of less than 20% to the target sequence to aid the prediction by applying a local bias (Davtyan *et al.*, 2012 ▸). Again, if closer homologues exist they should be used in practical applications.

The coevolutionary term is based on the predicted co­evolutionary data from other servers including *Gremlin* and *RaptorX-contact* (Wang, Sun *et al.*, 2017 ▸; Ovchinnikov *et al.*, 2014 ▸). Both algorithms predict contact pairs based on a statistical model that captures both the conservation and co-evolution patterns of a protein family.

The starting structures for annealing were built using *MODELLER* when a distant homolog was found using *HHpred*. Otherwise, the structure was initially generated by *PyMOL* as an extended structure. Next, 20 parallel simulated-annealing jobs were performed from 400 to 200 K in four million steps with different initial velocity seeds. The lowest *AWSEM* energy frame in each job was chosen for further trimming.

### 2.3. Trimming the predicted structure based on the r.m.s.f. value of the trajectory   

The root-mean-square fluctuation (r.m.s.f.) quantifies the fluctuations of the residues in a folded macromolecule. The r.m.s.f. also indicates the flexibility displayed by a protein throughout a simulation trajectory. The r.m.s.f. of a protein can be calculated from a molecular-dynamics trajectory using the deviations of the position in each frame from the average position over the whole trajectory. The *B* factor can be calculated from the relation of Kuzmanic & Zagrovic (2010 ▸) from the r.m.s.f. for each residue, 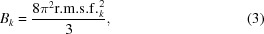
where *B_k_* and r.m.s.f.*_k_* are the corresponding *B* factor and r.m.s.f. value of the given residue *k*. Since the simulation protocol involves annealing the temperature, we calculated the r.m.s.f. using only the last million steps, which ensures that the motions have reasonably equilibrated. Finally, all residues that have an r.m.s.f. over 1.5 Å are trimmed from the phasing exploration rather than being simply reweighted. This value of the cutoff delineates the most flexible coil regions. These flexible regions are generally detrimental to finding accurate phases in molecular replacement.

### 2.4. Molecular replacement and automatic model refinement   

The *Phaser* program under the *Phenix* collective software platform (version 1.16-3549) was used for molecular replacement in the MR_AUTO mode (McCoy *et al.*, 2007 ▸; Liebschner *et al.*, 2019 ▸). *Phaser* was given 1.5 Å as an initial estimate of the r.m.s. error of the search model. The best solution from *Phaser* was used as input to *phenix.autobuild* for automatic model construction in its default mode (Terwilliger *et al.*, 2008 ▸). For PDB entry 1es5, we used *SHELXE* to rebuild a polyalanine backbone followed by *ARP*/*wARP* to further rebuild, refine and add side chains (Langer *et al.*, 2008 ▸; Thorn & Sheldrick, 2013 ▸). The log-likelihood gain (LLG) and the translation-function *Z*-score (TFZ) were then used to evaluate the molecular-replacement solutions. LLG indicates how much better the solution is compared with a random solution and the TFZ score indicates by how many standard deviations the LLG value from the solution of the translation search exceeds the mean LLG value from a set of random translations (McCoy *et al.*, 2007 ▸). The criterion for success for a trial was defined by the *R*_free_ value being less than 0.45 after model building. This value usually indicates a correct coordinate refinement based on a molecular-replacement solution. The *R* value measures how well the simulated diffraction pattern matches the experimentally observed diffraction pattern. The *R*_free_ value is the *R* value that comes from 5–10% of the experimental data that were not used in the refinement itself (Kleywegt & Jones, 1997 ▸; Brünger, 1992 ▸). This criterion is more stringent than that used in a paper describing molecular replacement based on *I-TASSER-MR* (Wang *et al.*, 2016 ▸). We have found quite a few cases where *Phaser* finds incorrect molecular-replacement solutions despite very high TFZ scores (≥8).

### 2.5. Metrics for evaluating structure prediction and molecular replacement   

Three metrics are used to evaluate the accuracy of the structure predictions: r.m.s.d., *Qw* and GDT-TS. R.m.s.d. describes the root-mean-square deviation of all atoms between the predicted and native structures when ideally aligned. R.m.s.d. was calculated by *PyMOL* version 2.0. *Qw* is a similarity metric given by the following equation (Eastwood *et al.*, 2001 ▸): 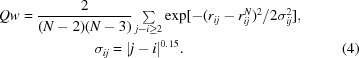
GDT-TS (Global Distance Test Total Score) is defined as the percentage of C^α^ atoms falling within distances of 1, 2, 4 and 8 Å. These four scores are then added up and divided by four. The map CC (map correlation coefficient) value is introduced to calculate the similarity between the two electron-density maps that are generated from the models or the X-ray data.

### 2.6. Calculating the TFZ score for pre-aligned structures   

To test whether the phasing search algorithm performance was suboptimal, we aligned the models predicted by *AWSEM-Suite* with the crystal structure in *PyMOL* using the cealign command. This aligned structure was then used to calculate the TFZ score using the MR_RNP mode in *Phaser* with the TRANSLATE VOLUME AROUND FRACTIONAL POINT 0 0 0 RANGE 0.1 command, except for those belonging to space group *P*1. For proteins with space group *P*1, we use the normal protocol in *Phaser* to calculate TFZ. The command get_cc_mtz_mtz in the *Phenix* package was then used to calculate the map CC value between the 2*mF*_o_ − *DF*_c_ map calculated from the PDB files and the σ_A_-weighted density map generated by *Phaser*.

## 3. Results   

### 3.1. *AWSEM-Suite* outperforms *I-TASSER-MR* and *AWSEM-Template* in solving the phase problem   

**Figure 2:**
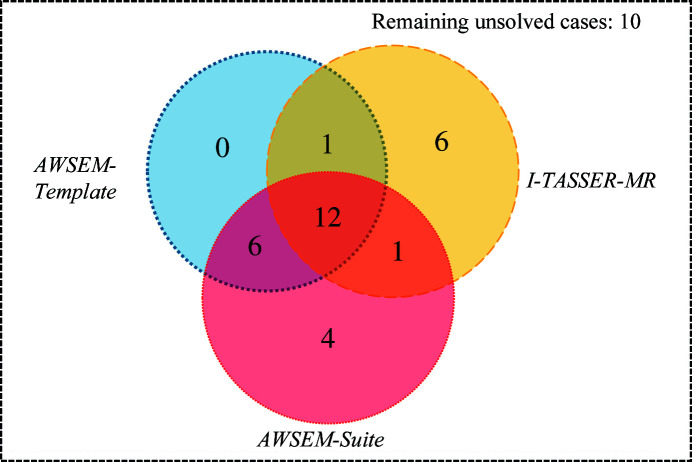
Venn diagram of the performance of *AWSEM* and *I-TASSER-MR* for 40 selected structures. The number in each section represents the number of cases solved by the corresponding protocol. *AWSEM-Suite* outperformed *I-­TASSER-MR* for ten cases in the data set, which enhances the overall success rate by 15% if both methods are used.

To better understand the performance of the structures predicted by *AWSEM-Suite* in solving the crystallographic phase problem, we chose a test set of 40 cases. To benchmark against existing methods, we chose 20 successful cases and 20 failed cases from the top of the list in Supplementary Tables S4 and S6 in the *I-TASSER-MR* paper by Wang *et al.* (2016 ▸), and we refer to this set as the ‘High-Res set’. The details for each case can be found in Supplementary Spreadsheet S1. This test data set was chosen for study as its members have a pairwise sequence-identity maximum of 25%, a sequence length of <300 residues and a resolution of ≤1.5 Å. Despite major differences in the two philosophical approaches to predicting structures, *AWSEM-Suite* performs well, modestly outperforming *I-TASSER-MR* for this data set. *AWSEM-Suite* uses a physics-based bioinformatics-biased molecular-dynamics simulation to find the lowest energy structure, while *I-TASSER-MR* searches the whole database and applies threading and averaging over multiple homolog models. For the selected set of 40 structures, *AWSEM-Suite* generated models that successfully solved 23 phasing targets. This is 15% more than the rate of solution by *I-TASSER-MR*. A user can achieve an overall 75% success rate by using both methods if one of them fails. *AWSEM-Suite* also performed better than a previous algorithm of ours, *AWSEM-Template*, as shown in Fig. 2 ▸. Ten of the cases solved by *AWSEM-Suite* failed in the *I-TASSER-MR* trial, so that an overall 25% higher success rate is achieved if both methods are used. The average sequence identity of the original template to the target sequence used in these cases was only 21.9%. This result demonstrates that *AWSEM-Suite* can generate a sufficiently reliable predicted model for molecular replacement and further coordinate refinement based on X-ray data. The average fraction of the structure for which a molecular model could be built automatically was 74.1%, which is similar to the result from *I-TASSER-MR*. A summary of the molecular-replacement trials and the corresponding structure-similarity scores comparing the crystal structures for both successful and failed cases is provided in Supplementary Spreadsheet S1.

**Figure 3:**
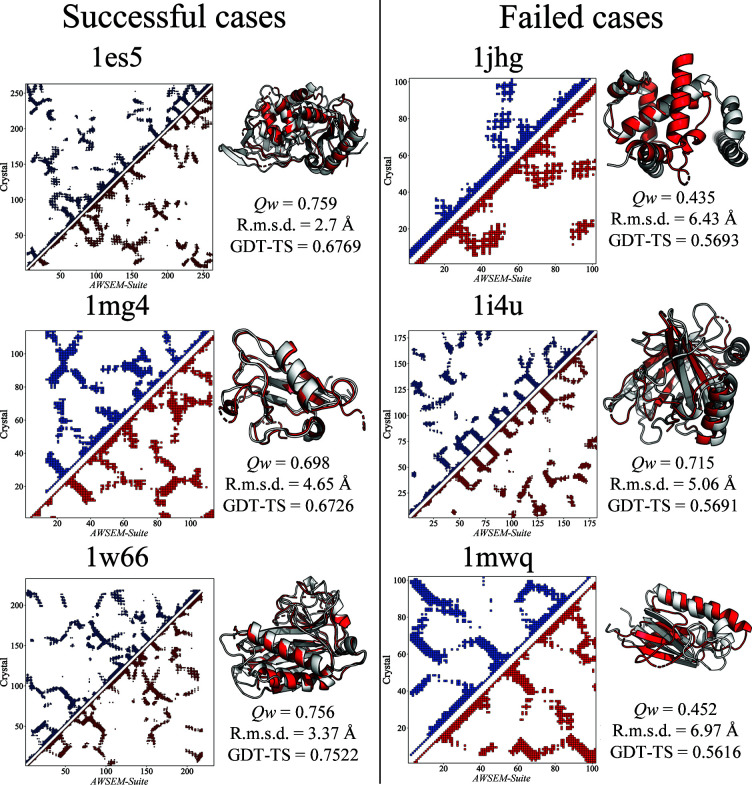
Prediction quality of six structure-prediction targets obtained using *AWSEM-Suite* in the High-Res data set. The contact map in the left of each panel allows comparison of the close contacts in the model and the crystal structure. The red squares correspond to amino-acid contacts in the crystal structure, while the blue squares correspond to those found in the *AWSEM-Suite*-predicted structure. The cutoff distance for forming a contact between C^α^ atoms has been set to 9.5 Å. The alignments of the *AWSEM-Suite* structure that lead to the trial with the lowest *R*_free_ value with the corresponding deposited crystal structures are shown on the right of each panel. The best predicted structures are shown in red, while the corresponding deposited crystal structures are shown in white.

The *AWSEM-Suite*-predicted structures for six representative cases from the data set are shown in Fig. 3 ▸. Among these examples, PDB entries 1jhg, 1i4u and 1mwq were failed cases, while PDB entries 1es5, 1mg4 and 1w66 represent successful cases. There is a clear gap in the GDT-TS value between failed and successful cases. Failed cases usually have a GDT-TS of >0.6, but the GDT-TS score for most successful cases is 0.65 or greater. It is also interesting that achieving a successful molecular-replacement solution does not require models with explicit side chains. Once the main chain is formed correctly, the side chains can be packed into a stable position. The six structures that *AWSEM-Suite* was able to solve but for which *I-TASSER-MR* was unsuccessful have original template sequence identities that range from 13% to 27%. For PDB entry 1w66, which has a sequence identity with the original template of only 13%, *AWSEM-Suite* yielded a very good model with an r.m.s.d. value around 1.3 Å, showing that molecular-dynamics simulation can overcome some energy barriers that pure informatics-based methodologies cannot. We note that *AWSEM-Suite* prediction is faster than the other protocols such as *I-TASSER-MR* and *MR-ROSETTA*. The average time for phasing using *AWSEM-Suite* for these cases was around 40 h, while the average time for *I-TASSER-MR* to converge was 63 h and that for *MR-ROSETTA* was even longer (Wang, Virtanen *et al.*, 2017 ▸).

A comparison of the density maps for the refined predicted structures and the crystal structures is provided in Supplementary Fig. S1. These maps indicate that although the predictions from *AWSEM-Suite* are coarse-grained, the optimal fit of a predicted main-chain structure to its corresponding crystal structure suffices to find a correct solution. Coordinate rebuilding and refinement programs based on X-ray data such as *phenix.autobuild* can recover the correct orientation and position for most of the side-chain atoms.

### 3.2. Statistical analysis of solved and failed cases with their deposited structures   

**Figure 4:**
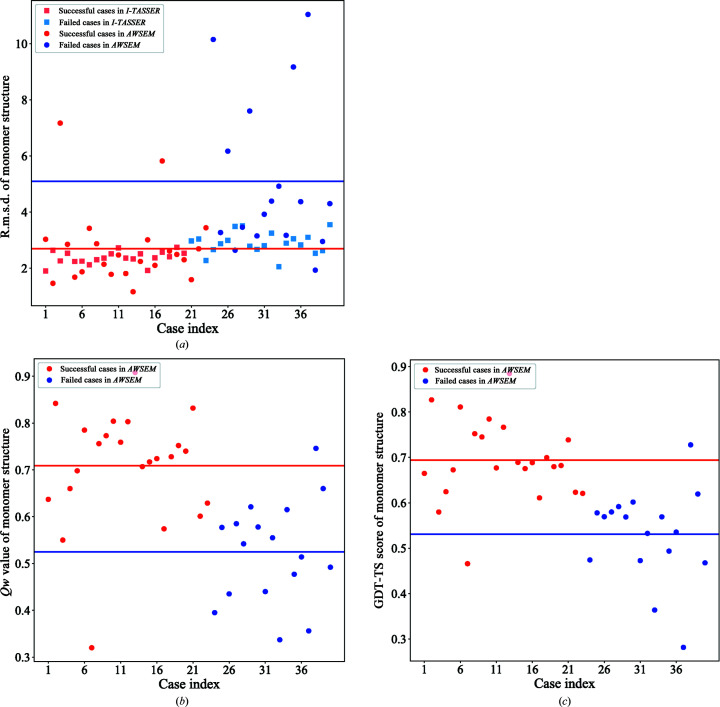
Summary of three different measures of prediction quality over all 40 structures that were investigated. The red and blue lines show the average values among successful cases and failed cases, respectively. A clear gap between the two lines is apparent. The exception to this rule, PDB entry 1rg8, is specifically discussed in the main text.

The r.m.s.d., *Qw* and GDT-TS values for the predicted monomer structure for both successful and failed cases are plotted in Fig. 4 ▸. The average r.m.s.d. value of the prediction for successful phasing cases from *AWSEM-Suite* was 2.69 Å, while the average r.m.s.d. of the prediction for failed phasing cases from *AWSEM-Suite* was 5.09 Å. These can be compared with what is seen for *I-TASSER-MR*-predicted results, where the r.m.s.d. value averaged over all successful cases was 2.38 Å but the average r.m.s.d. value for failed cases was 2.90 Å. Thus, we found that the molecular-replacement method is more tolerant of even less accurate coarse-grained models. The *Qw* value and GDT-TS scores show much clearer gaps between successful and failed cases, as illustrated in Fig. 4 ▸. The average *Qw* value for successful cases from *AWSEM-Suite* is 0.708, while it is 0.525 for failed cases from *AWSEM-Suite*. The average GDT-TS score for successful cases in *AWSEM-Suite* is 0.694, while that for failed cases in *AWSEM-Suite* was 0.53.

There is one outlier with respect to this pattern: PDB entry 1rg8. This protein has a very accurately predicted structure but nevertheless failed to give a sufficiently good phasing solution. For this target, the average r.m.s.d. value over the 20 predicted structures after trimming is 1.877 Å, which would indicate a quite accurate model of the portion that remains after trimming. The backbones of the predictions turn out to be very similar to the actual crystal structure, but the molecular-replacement solutions were not able accurately to place the copies of the protein in the unit cell (two per asymmetric unit in the crystal). To diagnose the problem in the molecular-replacement search, we aligned the *AWSEM-Suite*-predicted structures with the known crystal structure positions using *PyMOL* and then saved them as input for *phenix.autobuild*. The pre-aligned structures then generated successfully traced molecular models in 12 out of 20 trials, indicating that the predicted structure itself was sufficiently accurate but that the maximum-likelihood-based searching procedure was unable to find the molecular-replacement solution. We speculate that the separation of the rotation and translation searches in the molecular-replacement algorithm may cause the failure to find the appropriate minima in the whole six-dimensional molecular-replacement space when the number of copies in the unit cell is greater than 1.

We also examined whether there was any dependence of the probability of achieving a successful solution on the length of the sequence, the overall Structural Classification of Proteins (SCOP) class or the solvent content of the crystal as possible factors in determining the success of molecular replacement in Supplementary Fig. S2. SCOP classifies the structures based on the overall secondary structure. The longest monomers that we examined were in the 231–270 amino-acid range. *AWSEM-Suite* is more successful in yielding models that are capable of finding the correct molecular-replacement solution with larger sized proteins. Surprisingly, the highest rate of success for *AWSEM-Suite* was seen for α/β folds. A previous report suggested that all-α folds are more often phased successfully using computationally predicted structures during molecular replacement (Bibby *et al.*, 2012 ▸). We also found that there is generally a strong correlation between the percentage solvent content of the crystal structure and the TFZ score. This correlation indicates that a higher solvent content gives a stronger signal in the unit cell and makes it easier to find phase information.

### 3.3. *AWSEM-Template* can give accurate predictions when the coevolutionary input information is not correct   

**Figure 5:**
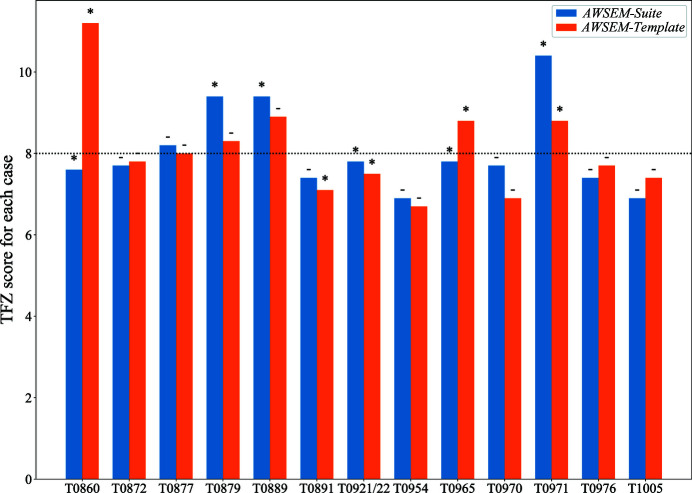
Comparison of the performance of *AWSEM-Suite* and *AWSEM-Template*. The *y* axis shows the TFZ score of the best predicted structure in each case. A star at the top of the bar represents a successful case, while a dash represents a failed case. *AWSEM-Suite* has six successful structures, while *AWSEM-Template* has only five. T0879 and T0889 were only solved by *AWSEM-Suite*, but another case, T0891, was only solved by *AWSEM-Template*.

To complement the tests that we have just described, we chose another data set from the CASP12 and CASP13 competitions. The cases in this data set are those examples with homologs that have only low sequence-identity templates (less than 30%) and a monomer length between 90 and 400, where the number of noncrystallographic equivalent copies in the unit cell is less than four and also, of course, where the experimental X-ray data were available when the prediction was performed. With such restraints, eight targets from CASP12 and six targets from CASP13 were selected. They are T0860, T0872, T0877, T0879, T0889, T0891, T0921, T0922, T0954, T0965, T0970, T0971, T0976 and T1005. A detailed analysis of the quality of prediction and molecular replacement is shown in Supplementary Spreadsheet S2. Among these examples, T0921 and T0922 form a heterodimer (PDB entry 5m2o) and were phased using the predicted structures of both components. No proteins in the CASP12/13 data set were homologous to any of the structures in our High-Res set. We compared the two versions of *AWSEM* for these 13 cases. We note that we used the protein sequences provided by the CASP group; these are sometimes several residues longer than the deposited crystal structure sequences in the PDB. The predicted contact list for the coevolutionary term came from either the *Gremlin* server (for the CASP12 cases) or the *RaptorX-contact* server (for the CASP13 cases). Both of these servers used databases that were generated before the start of the corresponding CASP competition, thus ensuring that the selection provides blind tests for prediction (Ovchinnikov *et al.*, 2014 ▸; Xu, 2019 ▸). Fig. 5 ▸ shows that *AWSEM-Suite* yielded two cases (T0879 and T0889) that outperformed *AWSEM-Template*. T0891 is a case, however, where *AWSEM-Template* succeeded in yielding good phases while the *AWSEM-Suite* prediction failed.

*AWSEM-Suite* combines both template and coevolutionary information with the physico-chemical energy terms of *AWSEM*. To better understand why *AWSEM-Template*, which does not include coevolutionary information, would out­perform *AWSEM-Suite*, we analyzed whether the input of coevolutionary information used to predict PDB entry 1unq and T0891 was in conflict with the other input terms. For PDB entry 1unq, with 125 residues, there were a total of 120 hits in the *Gremlin*-predicted coevolutionary contact list which had an assigned probability over 0.5. 67 of these contacts were correctly present in the crystal structure. Only 39% of these predicted contacts were present in the template structure. For T0891, the percentage of the predicted contacts that were present in the template structure was 45%. These lower percentages indicate that there are conflicts between the coevolutionary data and the template structure, which could explain the relatively poor performance of the hybrid model for these targets.

### 3.4. Successful application of *AWSEM-Suite* to previously unphased targets   

We used *AWSEM-Suite* to phase two previously unsolved structures. For one of these proteins, RuHACL (PDB entry 6xn8), we ran both *AWSEM-Suite* and *AWSEM-Template* starting from the known protein sequence. The template chosen for the initial homolog term was PDB entry 5dx6, which has only 24% sequence identity to the query sequence. The *Escherichia coli* and *Oxalobacter formigenes* oxalyl-CoA decarboxylases are 38–40% identical to RuHACL, but we excluded these structures from fragment generation in our protocol in order to evaluate the performance when only distant homologs are available. Using chain *A* of PDB entry 5dx6 in a molecular-replacement search gave a TFZ score of 5.4 and an *R*_free_ value of 0.525 after *phenix.autobuild*, where both values generally indicate a failed solution. Among the *AWSEM*-predicted structures, 14 of the 20 trials obtained from *AWSEM-Suite* were successfully able to solve this case, while none of the *AWSEM-Template* trials could solve the structure even though multiple trials yielded a TFZ score as high as 14. The *R*_free_ values of these successful trials ranged from 0.224 to 0.366. We also tried to solve the RuHACL structure using the *I-TASSER-MR* online server, but again no solution was found. The five top output results all yielded *R*_free_ values of around 0.55.

The other example is the PEX4–PEX22 complex (PDB entry 6xod). In this case, the best original template itself can solve the major part of the structure independently, but the model from *AWSEM-Suite* did help to build the position of a helix that the initial phasing from the template-generated model could not rebuild. These cases indicate that *AWSEM-Suite*-predicted structures can indeed aid in phasing novel crystal structures.

### 3.5. Trimming structures enhances the signal from the correctly predicted regions   

Truncation of the model has been proven to be useful in many molecular-replacement protocols. The key is learning how to pick out those regions which have the largest fluctuations or that are most likely to be in error. In our pipeline, r.m.s.f.-based truncation proved useful in most cases. We analyzed the secondary structure of the trimmed region in the lowest *R*_free_-value trial among all 23 successful molecular-replacement cases in the High-Res data set. Three secondary-structure types (helix, strand and coil) were specified by *DSSP* from the PDB structure. The regions which are not shown in the crystal structure are termed ‘unstructured residues’ (Dunker *et al.*, 2000 ▸). The most frequently truncated residues are found in coil regions (46.75%), followed by those in the unstructured region (32.67%) and then those in helical regions (14.99%); the least often truncated are found in strand regions (5.59%). These values indicate that truncation can successfully locate the most variable regions in the structure, which are usually the least accurate in the original model. We note that the definition of the r.m.s.f. value is similar to that of the AVS value in the *I-TASSER-MR* paper (Wang *et al.*, 2016 ▸), but the strategies for truncation are different.

### 3.6. The correct estimation of *B* factors leads to better solutions   

**Figure 6:**
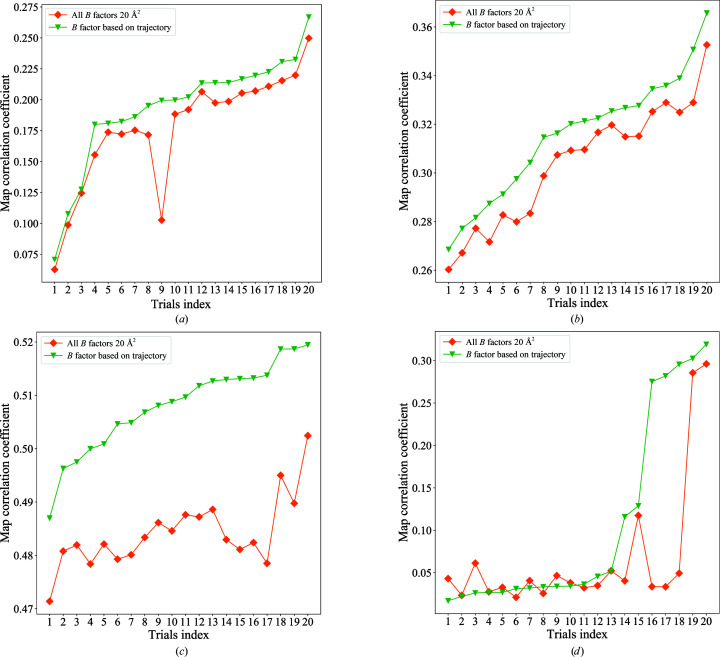
Rationale of the *B* factor in predicting (*a*) PDB entry 1es5, (*b*) PDB entry 1i8o, (*c*) PDB entry 1w66 and (*d*) PDB entry 1z0w. A uniform value of 20 Å^2^ and a calculated *B* factor based on the molecular-dynamics trajectory are applied to the same predicted models for molecular replacement. The orange diamonds represent all *B* factors assigned as 20 Å^2^, while the green triangles represent structures with *B* factors calculated from the trajectory.

The *B* factor or temperature factor is used to describe the reduction of X-ray scattering or coherent neutron scattering caused by local thermal motions. This factor is related to the isotropically averaged mean-square displacement of each atom. Several papers have discussed the effects of *B* factors on molecular replacement (Keegan *et al.*, 2015 ▸; Read & Chavali, 2007 ▸). Decreasing the *B* factors for the hydrophobic core of the protein while increasing the *B* factors for the surface-exposed residues often improves the success rate of molecular replacement (Lebedev *et al.*, 2008 ▸). Several ways of including *B* factors have been summarized in previous publications (Li & Brüschweiler, 2009 ▸). One approach is to simply add a uniform *B* factor for the whole structure. Another method is to employ a *B* factor for each amino acid. Using an accurate *B* factor greatly helps the likelihood of finding a good molecular-replacement solution for four selected cases. We tested two *B*-factor assignment schemes, as shown in Fig. 6 ▸. Firstly, we kept the coordinates of each atom the same in the predicted model but set the *B* factor of all atoms to 20. We calculated the map CC value between the deposited structure and the map derived from these models placed by *Phaser* and compared them with the map CC for *Phaser*-placed models with the same starting coordinates but different *B*-factor estimates. The results showed there is a significant improvement of the map CC value when one uses the r.m.s.f.-based *B* factor rather than a uniform value of 20 Å^2^. For PDB entry 1z0w, we found several trials that had a significant enhancement of the map CC value from around 0.05 to 0.3. For a failed search the map correlation coefficient is usually less than 0.1, while a correct solution usually gives a value higher than 0.25. We found that better estimation of the *B* factors helped the molecular-replacement program to find a successful phasing solution.

## 4. Discussion   

The results show that *AWSEM-Suite* has value in aiding molecular-replacement methods in crystallography. Our data reveal several key aspects of optimally using a predicted model for molecular replacement. Firstly, we have evaluated the effect of the overall structure quality on the success rate of molecular replacement. We have also demonstrated how truncations and *B* factors influence the search process.

To understand the role of cofactors in structure prediction, we also performed a frustration analysis of the models. Frustration is a term that describes the situation where a physical system is unable to simultaneously achieve minimum energies for each of its molecule subparts individually (Ferreiro *et al.*, 2018 ▸). The folding-energy landscape is biased toward its folded ensemble throughout the configuration space and therefore lacks deep kinetic traps that would otherwise frustrate folding (Chen *et al.*, 2019 ▸). There are often biological needs for this conflict with folding that need to be met in the specification of protein sequences. These include the necessity for interactions with other peptide chains or with small cofactor molecules which therefore change the frustration patterns of proteins. We have used *AWSEM-frustratometer*, an energy-landscape theory-inspired algorithm that aims to localize and quantify the energetic frustration present in protein molecules, in order to see where the frustration in the predicted protein structures lies (Parra *et al.*, 2016 ▸). The frustration index characterizes the distribution of a change in the energy of the native state where local alterations are made leading to structural decoys. These alterations may be made by mutation or conformational changes. Two examples of frustration patterns are shown in Supplementary Fig. S3 and indicate that *AWSEM-Suite*-predicted structures can be poor if they are predicted without including the explicit presence of small-molecule cofactors. For example, PDB entry 1tu9 is a close homolog of human hemoglobin, and including the heme decreased the frustration compared with that of the *AWSEM-Suite* force-field prediction made without the heme. A large number of highly frustrated interactions were observed in the empty heme-binding pocket. The relative positions of secondary structures are crucial to the success of molecular replacement. In the case of PDB entry 1tu9, all of the secondary-structure elements were well predicted, but without the cofactor, they formed a more compact structure than the actual native structure owing to the lack of cofactors in the prediction. This is familiar from NMR studies of apomyoglobin, for example (Jennings & Wright, 1993 ▸). Such structural changes between the holo and apo forms of a protein are quite expected. In the case of PDB entry 1mwq, the cofactor ZnCl_3_ binds to His24, Ser59 and Arg21 (Willis *et al.*, 2005 ▸). The absence of cofactors in the simulation model resulted in too short a distance between the two α-helices and the β-sheet to accommodate the cofactor.

**Figure 7:**
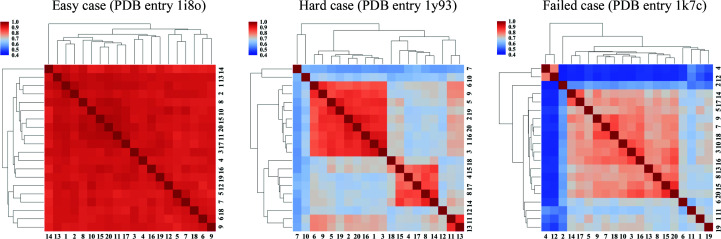
Clustergrams of three representative cases. Among these cases, PDB entry 1i8o is a easy case where 18 of 20 trials succeeded and PDB entry 1y93 is a hard case where only two of 20 trials succeeded. For PDB entry 1k7c, no trials succeeded.

How many predictions should be generated for a given blind case? We have found it informative to cluster the predicted structures based on their mutual *Q* value for the 20 independent runs in the 40 cases in the High-Res data set. Three representative clustergrams are shown in Fig. 7 ▸. For most of the easy targets (where more than half of the 20 trials led to a successful molecular-replacement solution), clustering yields a single huge cluster in which nearly every structure is similar to the others. If a target is hard to solve, such as PDB entry 1y93, where only two of the 20 trials succeeded, the *AWSEM-Suite* runs usually display several smaller clusters, with each cluster having a distinct fold. Only one or two of these small clusters correspond to the native basin. The clustering patterns for failed cases were similar to those for hard cases, but in these cases none of the clusters were found to work in phasing. These clusters tended to be more dissimilar from each other and the relative *Q* value between each of the small clusters can be as low as 0.5. In sum, if the clustergrams show a single huge cluster or several highly inter-similar clusters, more trials should be performed. In contrast, if the predictions are not clustered very well, this may indicate that the target itself is too hard for the protocol. All of the clustergrams for the 40 cases are shown in Supplementary Fig. S4.

The full molecular-replacement problem requires optimization in a six-dimensional space of translation and rotation. To accelerate the searching procedure, programs usually divide the search into separate rotation and translation steps, reducing the six-dimensional search to two three-dimensional searches. This splitting strategy speeds up the joint search but also has the chance of missing a correct solution. *Phaser* picks the highest hits from the rotation search after clustering as inputs for subsequent translation searches. We therefore devised a way to separate failures that arise from molecular-replacement search approximations from intrinsic limitations that depend on the quality of the predicted model. We aligned the predicted models with the crystal structures to check how many could then be rebuilt if they started from a correct molecular-replacement position in the unit cell. In this case a total of 27 cases were able to be successfully rebuilt starting from the *AWSEM-Suite*-generated structures. As Supplementary Fig. S5 demonstrates, among the four additional solved cases most trials of PDB entries 1i1j and 1rg8 are solvable, indicating that the search procedure in *Phaser* is apparently inadequate for these cases. PDB entries 1i4u and 1kq6 each had one trial that succeeded, indicating that the outliers in a clustergram sometimes represent a native-like basin. For PDB entry 1es5, most trials have a map CC value of over 0.25 with the crystal density map, indicating a possible correct solution, but all trials using *Phaser* failed. When we used *SHELXE* and *ARP*/*wARP* the same predicted structures often achieved successful solutions. Although the TFZ score evaluation from *Phaser* works well for most cases, there is still some space for it to improve in efficacy.

Schemes of model-based structural refinement using different technologies have recently been developed and tested in the recent CASP13 experiments. Some of these schemes have achieved very good results (Read *et al.*, 2019 ▸). Our group’s principal component-guided refinement scheme has been shown to be efficient in improving TFZ scores for several tested cases (Lin *et al.*, 2019 ▸). In this approach, the predicted coarse-grained structures are further refined based on the principal components of fluctuational motions calculated from an eight-million-step constant-temperature simulation using the coarse-grained *AWSEM* model. The principal component-guided simulations accelerate the sampling of protein conformational space to target structures that are close to the crystal structure. The application of these methods could further improve the quality of predicted structures for molecular replacement and could help to enhance the success rate of phasing through molecular replacement from predicted models. We also note that different X-ray refinement codes such as *Buccaneer* and *ARP*/*wARP* can show better performance for different X-ray data resolution ranges when compared with *phenix.autobuild* (Cowtan, 2006 ▸; Langer *et al.*, 2008 ▸). Combining such multiple approaches into a strategy to complement pure distant-homology molecular-dynamics simulation is important for the purpose of finding better molecular-replacement solutions.

The *AWSEM-Suite* algorithm has been implemented as an online server at https://awsem.rice.edu (Jin, Contessoto *et al.*, 2020 ▸). This server could benefit crystallographers at large, and the server documentation includes an example of how to use the server output to phase X-ray data in *Phenix*. We also note that *AWSEM-Suite* should be used in conjunction with other methods, as it often produces orthogonal models. As we have described, using a combination of multiple methods can maximize the probability of being able to solve a structure.

## Supplementary Material

Supplementary Spreadsheet S1. DOI: 10.1107/S2052252520013494/mf5047sup1.xlsx

Supplementary Spreadsheet S2. DOI: 10.1107/S2052252520013494/mf5047sup2.xlsx

Supplementary Figures. DOI: 10.1107/S2052252520013494/mf5047sup3.pdf
