# Supplementary material for: Molecular-replacement phasing using predicted protein structures from AWSEM-Suite
Source: IUCrJ. 2020 Oct 27;7(Pt 6):1168–78. doi: 10.1107/S2052252520013494 (PMC7642774; doi:10.1107/S2052252520013494)
Supplement: Supplementary file 3 [file m-07-01168-sup3.pdf]

IUCrJ

Volume 8 (2021)

Supporting information for article:

Molecular-replacement phasing using predicted protein structures from *AWSEM-Suite*

Shikai Jin, Mitchell D. Miller, Mingchen Chen, Nicholas P. Schafer, Xingcheng Lin, Xun Chen, George N. Phillips, Jr. and Peter G. Wolynes

Contents

S1 Electron density map comparison of 6 structure prediction targets corresponding in Figure 3. 2

S2 Evaluation the quality of molecular replacement with other quantities. 2

S3 Differential frustration analysis of 1tu9 and 1mwq. 3

S4 Clustermap for different targets. 4

S5 Map CC score for four cases that pre-aligned search of AWSEM-Suite predicted model can solve. 12

S1 Electron density map comparison of 6 structure prediction targets corresponding in Figure 3.

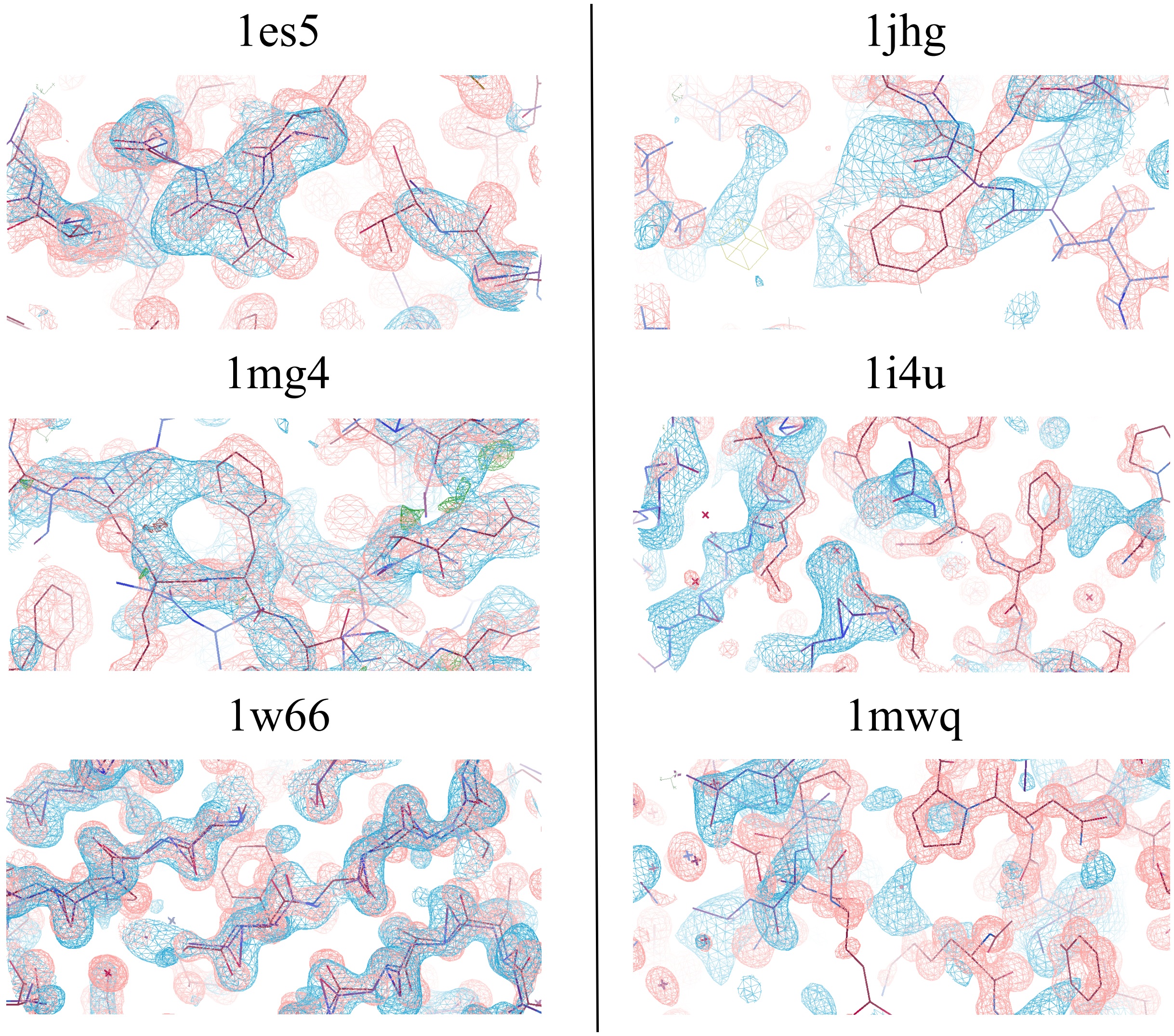

Figure S1: The electron density maps of 6 structure prediction targets corresponding in Figure 3. The red structure and density map show the data from crystal structure while the blue structure and density map show the data from AWSEM-Suite predicted structure after Phaser placement.

S2 Evaluation the quality of molecular replacement with other quantities.

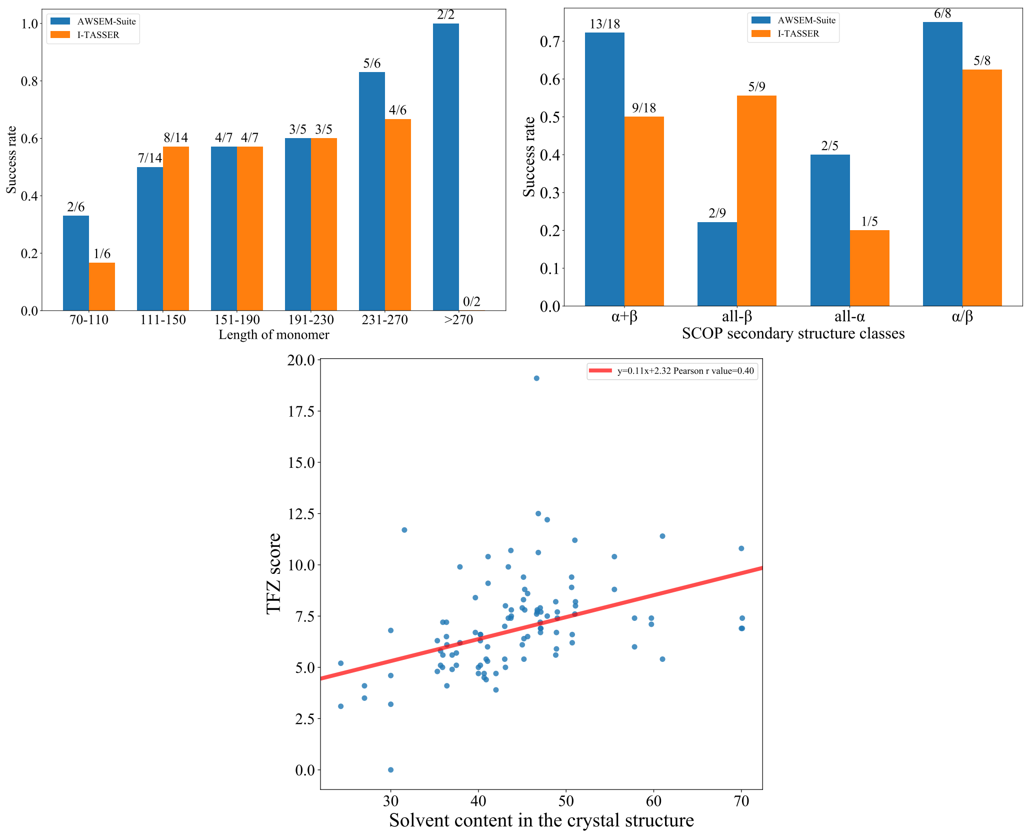

Figure S2: Evaluation the quality of molecular replacement with other quantities. The success rate of AWSEM-Suite as a function of the monomer length and secondary structure classes are plotted in the top and middle. AWSEM-Suite and I-TASSER are shown in blue and orange colors. The bottom is the TFZ score as a function of solvent content of the crystal structure (one outlier 1w66 is excluded). A strong correlation (Pearson r-value equals 0.4) is founded.

S3 Differential frustration analysis of 1tu9 and 1mwq.

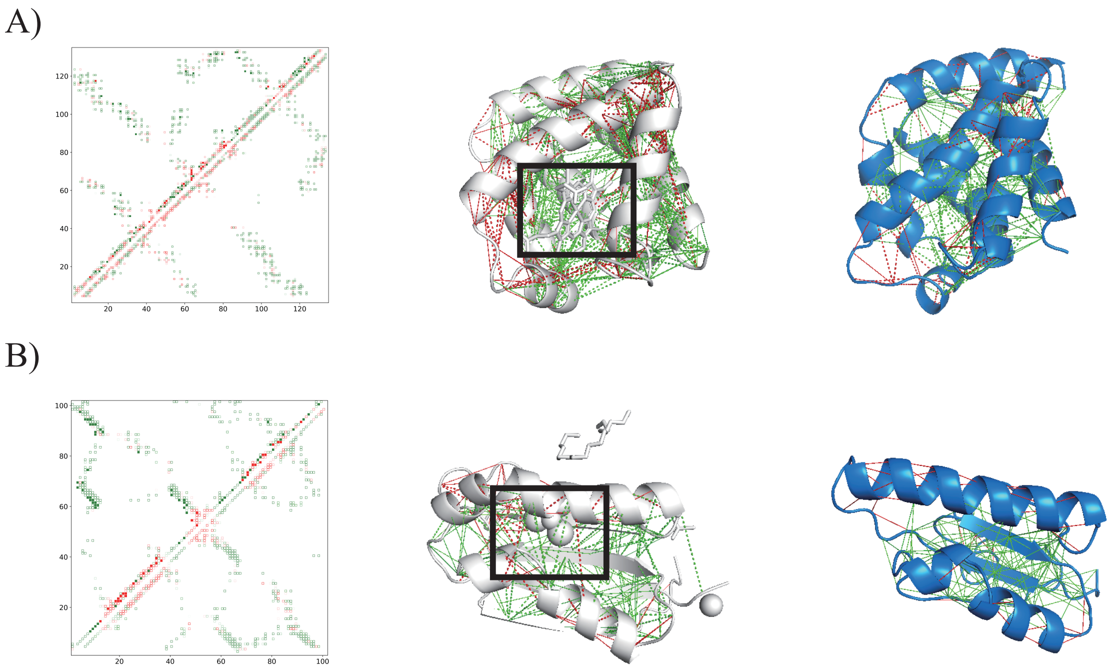

Figure S3: Differential frustration analysis of 1tu9 and 1mwq. The frustration contact maps in the left are comparing the residue frustration of the crystal structure (upper left) and the submitted structure (lower right). The color shows the range of frustration for each residue and the differential part of frustration analysis between two sturctures are plotted in green or red solid square (red solid square represents the residue interaction is a highly frustrated interaction in predicted structure but not in the crystal while the green square represents the a minimally frustration interaction is presents in predicted structure but not crystal). The structures on the right shows the frustration pattern of crystal (white) and predicted structure (blue). The position of cofactors are highlighted black box.

S4 Clustermap for different targets.

Figure S4: Clustermap for different targets. Among these targets, 1i8o, 1w66, 1f86, 1vdw, 1vkk are easy targets. The remaining solvable cases are all belong to hard targets.

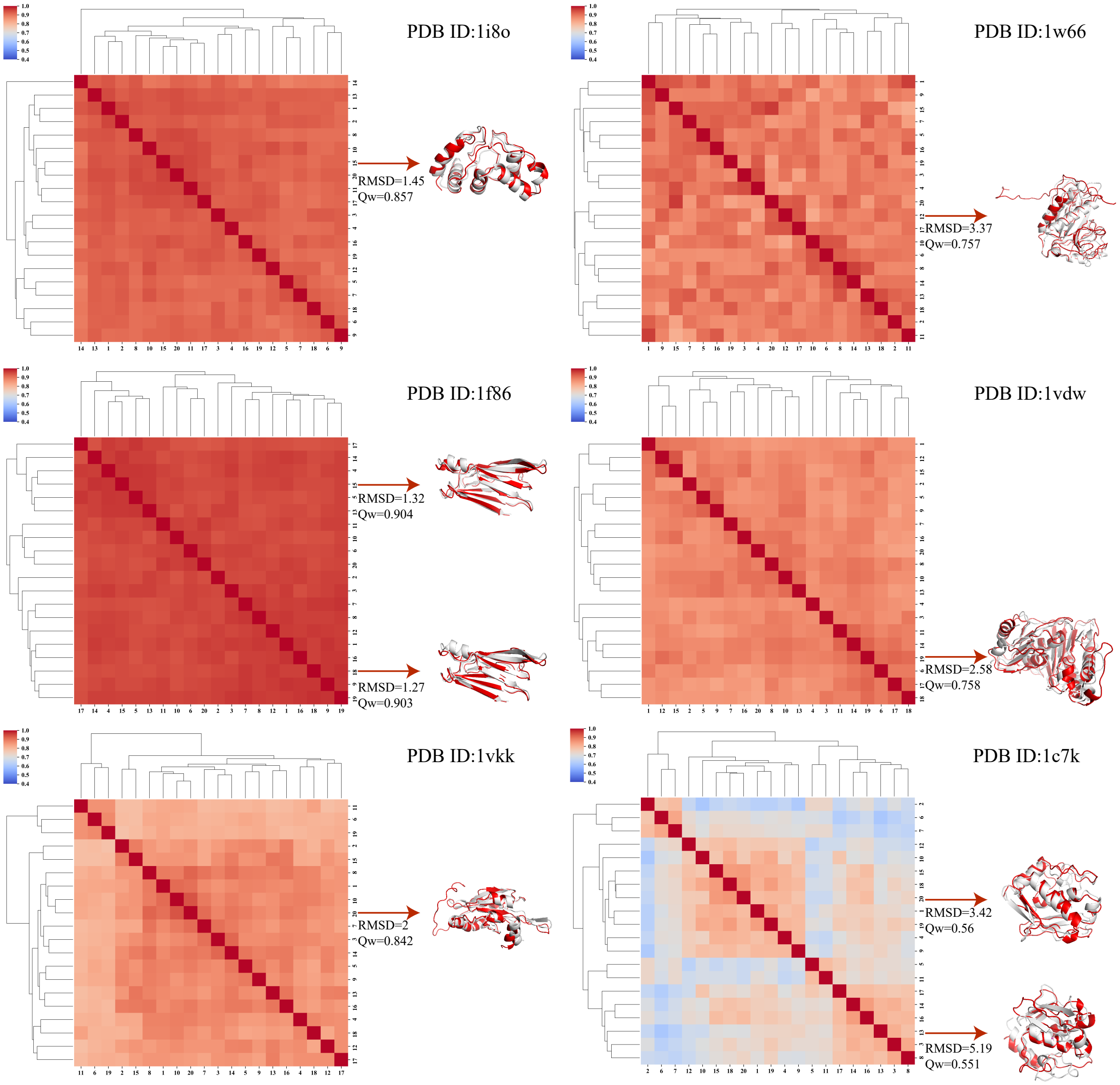

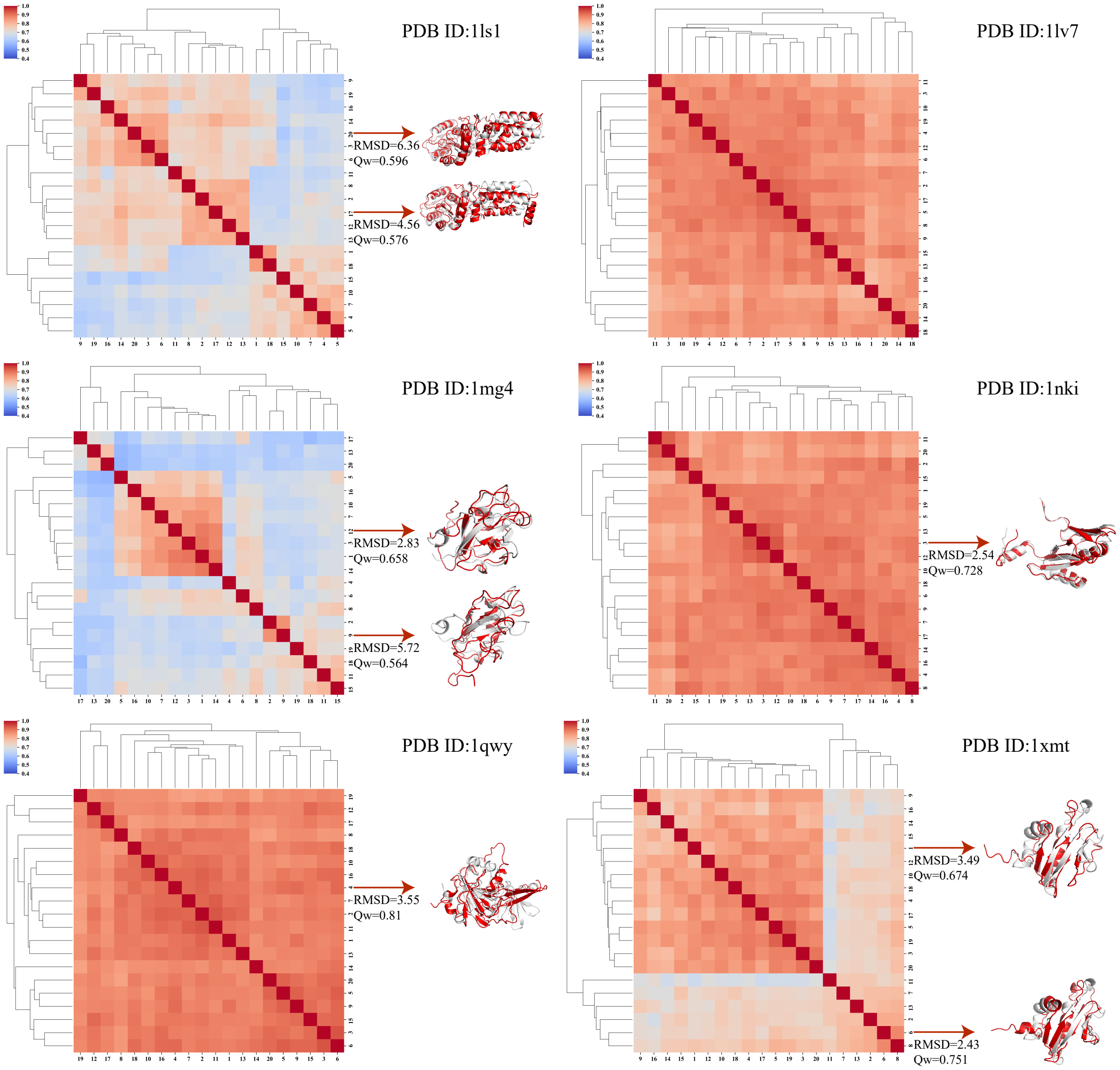
PDB ID:1ls1
RMSD=6.36
Qw=0.596
RMSD=4.56
Qw=0.576
PDB ID:1lv7
PDB ID:1mg4
RMSD=2.83
Qw=0.658
RMSD=5.72
Qw=0.564
PDB ID:1nki
RMSD=2.54
Qw=0.728
PDB ID:1qwy
RMSD=3.55
Qw=0.81
PDB ID:1xmt
RMSD=3.49
Qw=0.674
RMSD=2.43
Qw=0.751

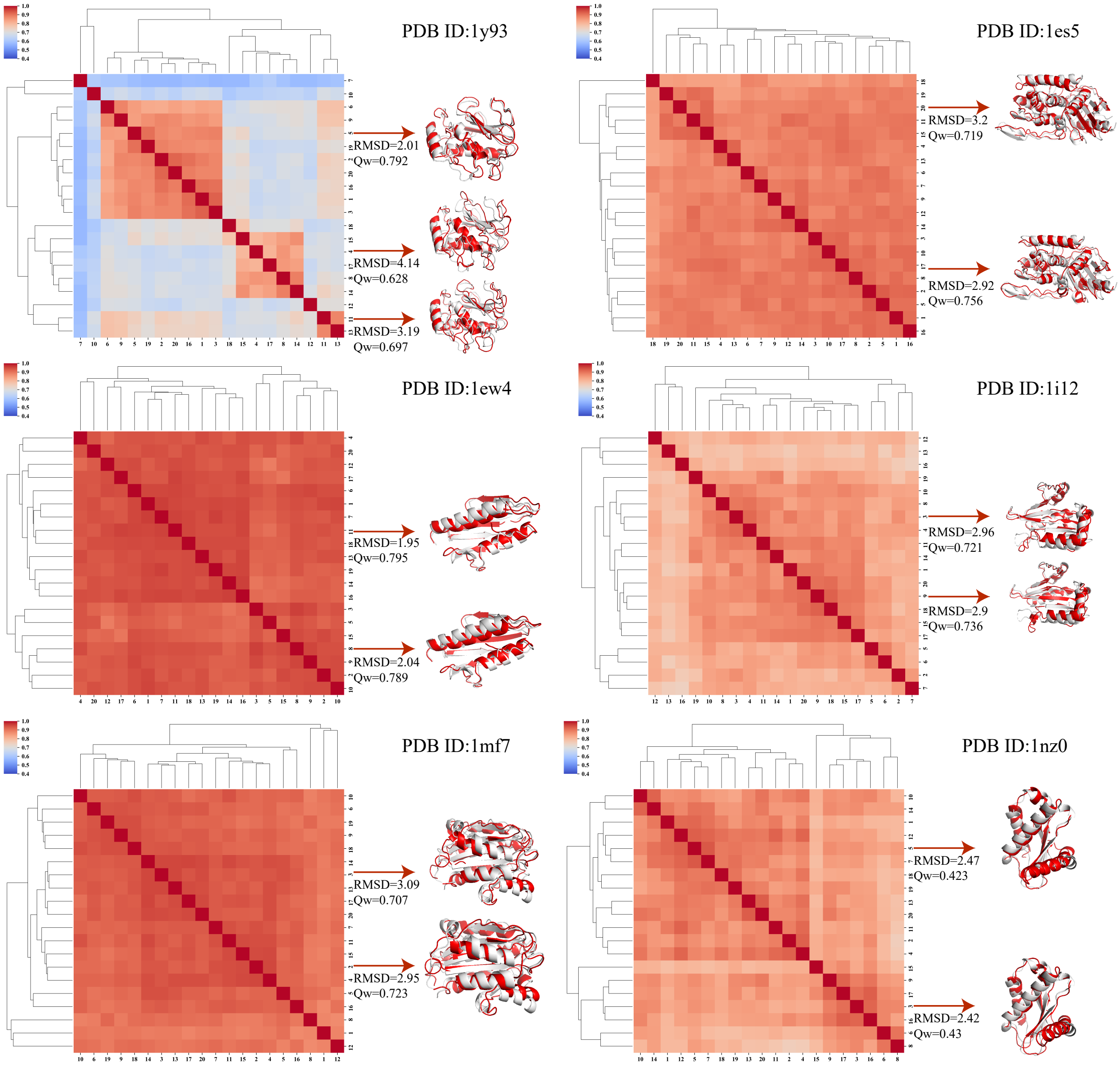

PDB ID:1y93
RMSD=2.01
Qw=0.792
RMSD=4.14
Qw=0.628
RMSD=3.19
Qw=0.697
PDB ID:1es5
RMSD=3.2
Qw=0.719
RMSD=2.92
Qw=0.756
PDB ID:1ew4
RMSD=1.95
Qw=0.795
RMSD=2.04
Qw=0.789
PDB ID:1i12
RMSD=2.96
Qw=0.721
RMSD=2.9
Qw=0.736
PDB ID:1mf7
RMSD=3.09
Qw=0.707
RMSD=2.95
Qw=0.723
PDB ID:1nz0
RMSD=2.47
Qw=0.423
RMSD=2.42
Qw=0.43

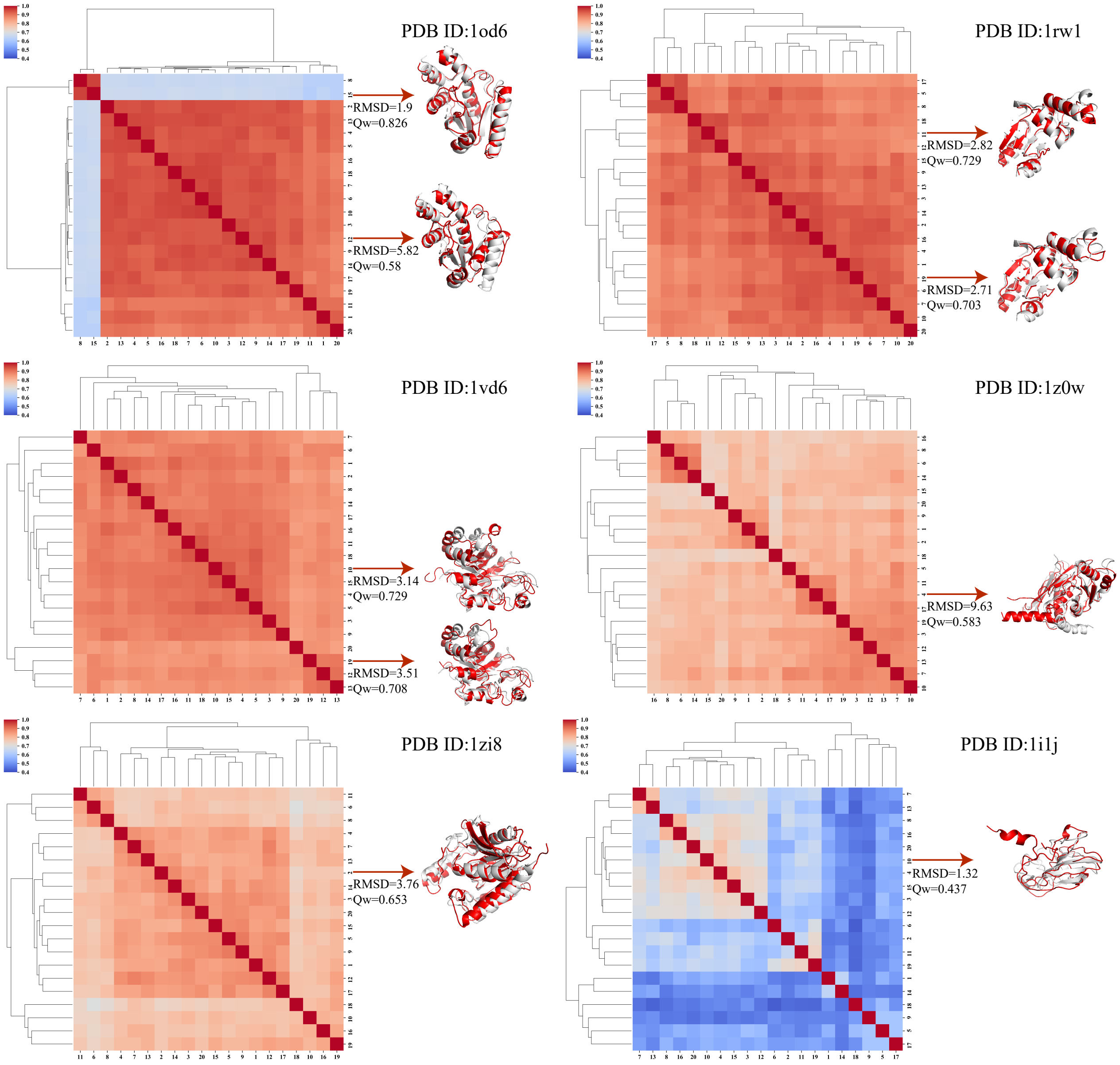

PDB ID:1od6
RMSD=1.9
Qw=0.826
RMSD=5.82
Qw=0.58
PDB ID:1rw1
RMSD=2.82
Qw=0.729
RMSD=2.71
Qw=0.703
PDB ID:1vd6
RMSD=3.14
Qw=0.729
RMSD=3.51
Qw=0.708
PDB ID:1z0w
RMSD=9.63
Qw=0.583
PDB ID:1zi8
RMSD=3.76
Qw=0.653
PDB ID:1i1j
RMSD=1.32
Qw=0.437

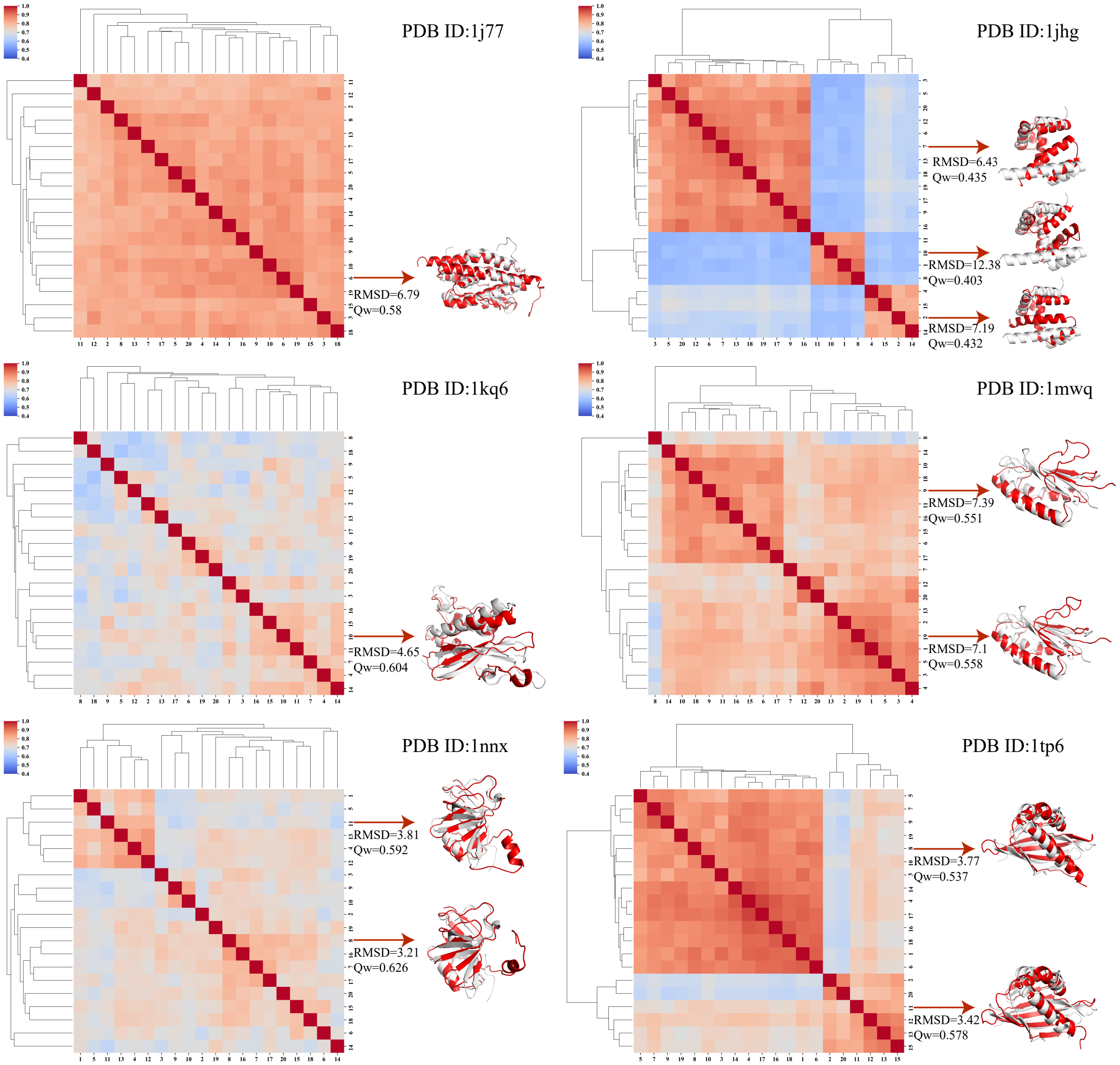

PDB ID:1j77
RMSD=6.79
Qw=0.58
PDB ID:1jhg
RMSD=6.43
Qw=0.435
RMSD=12.38
Qw=0.403
RMSD=7.19
Qw=0.432
PDB ID:1kq6
RMSD=4.65
Qw=0.604
PDB ID:1mwq
RMSD=7.39
Qw=0.551
RMSD=7.1
Qw=0.558
PDB ID:1nnx
RMSD=3.81
Qw=0.592
RMSD=3.21
Qw=0.626
PDB ID:1tp6
RMSD=3.77
Qw=0.537
RMSD=3.42
Qw=0.578

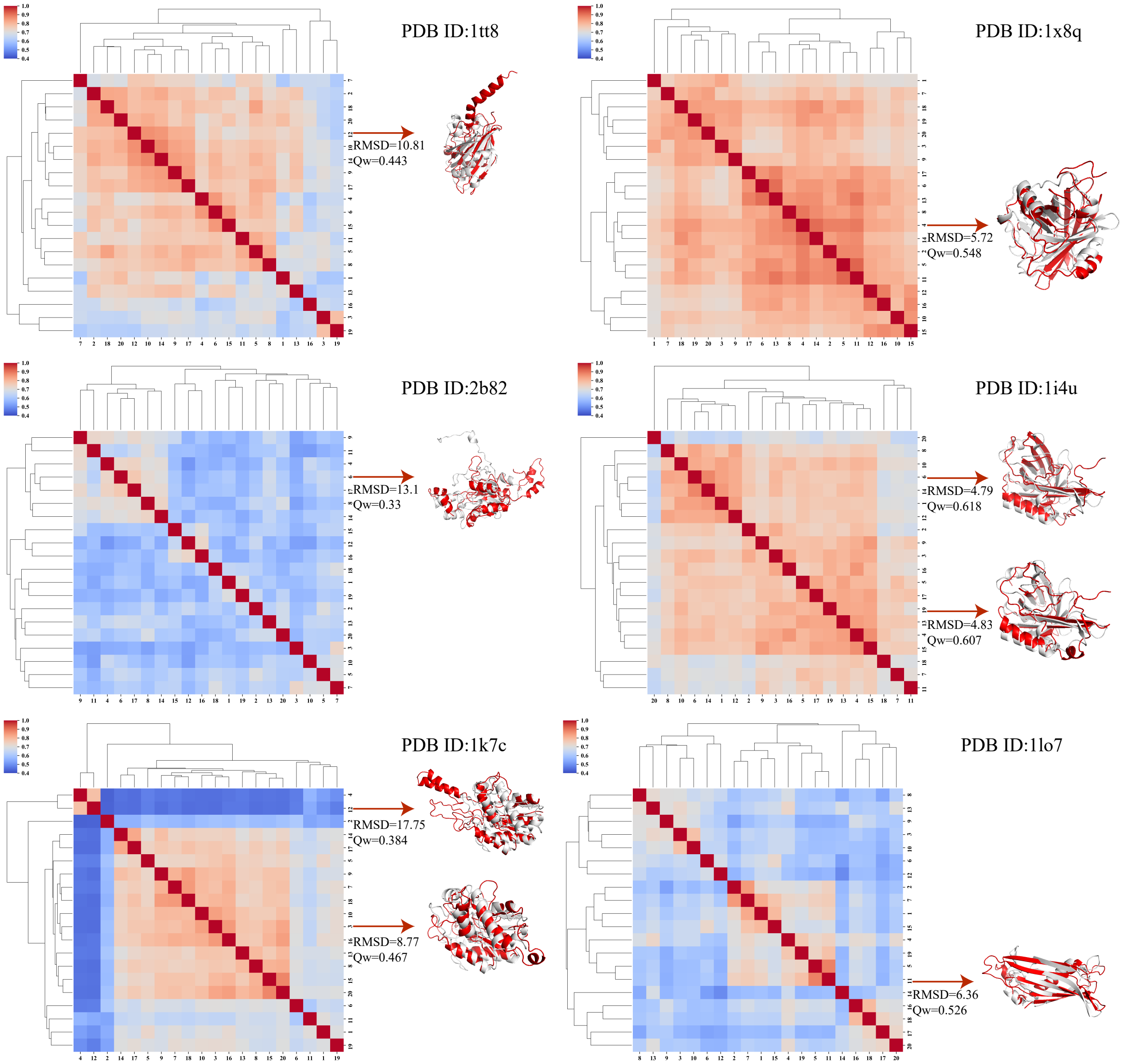
PDB ID:1tt8
RMSD=10.81
Qw=0.443
PDB ID:1x8q
RMSD=5.72
Qw=0.548
PDB ID:2b82
RMSD=13.1
Qw=0.33
PDB ID:1i4u
RMSD=4.79
Qw=0.618
RMSD=4.83
Qw=0.607
PDB ID:1k7c
RMSD=17.75
Qw=0.384
RMSD=8.77
Qw=0.467
PDB ID:1lo7
RMSD=6.36
Qw=0.526

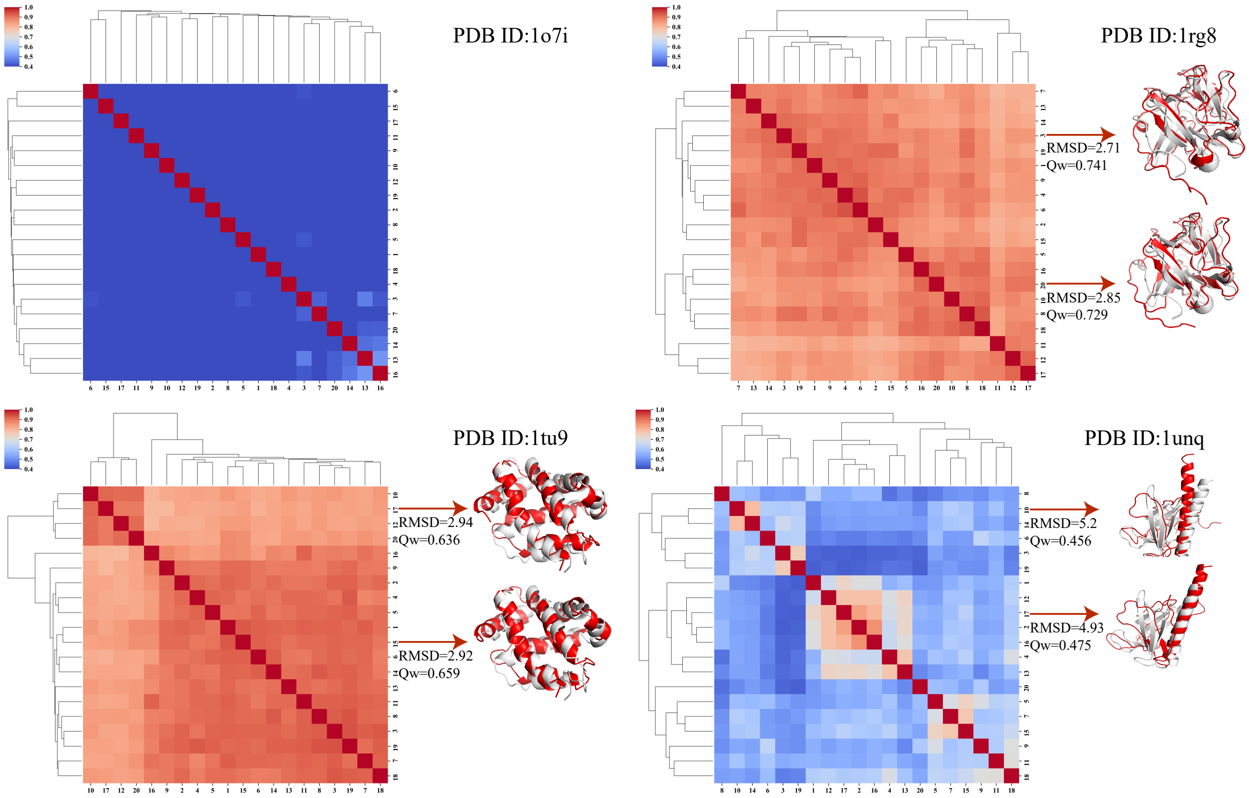

PDB ID:1o7i
PDB ID:1rg8
RMSD=2.71
Qw=0.741
RMSD=2.85
Qw=0.729
PDB ID:1tu9
RMSD=2.94
Qw=0.636
RMSD=2.92
Qw=0.659
PDB ID:1unq
RMSD=5.2
Qw=0.456
RMSD=4.93
Qw=0.475

S5 Map CC score for four cases that pre-aligned search of AWSEM-Suite predicted model can solve.

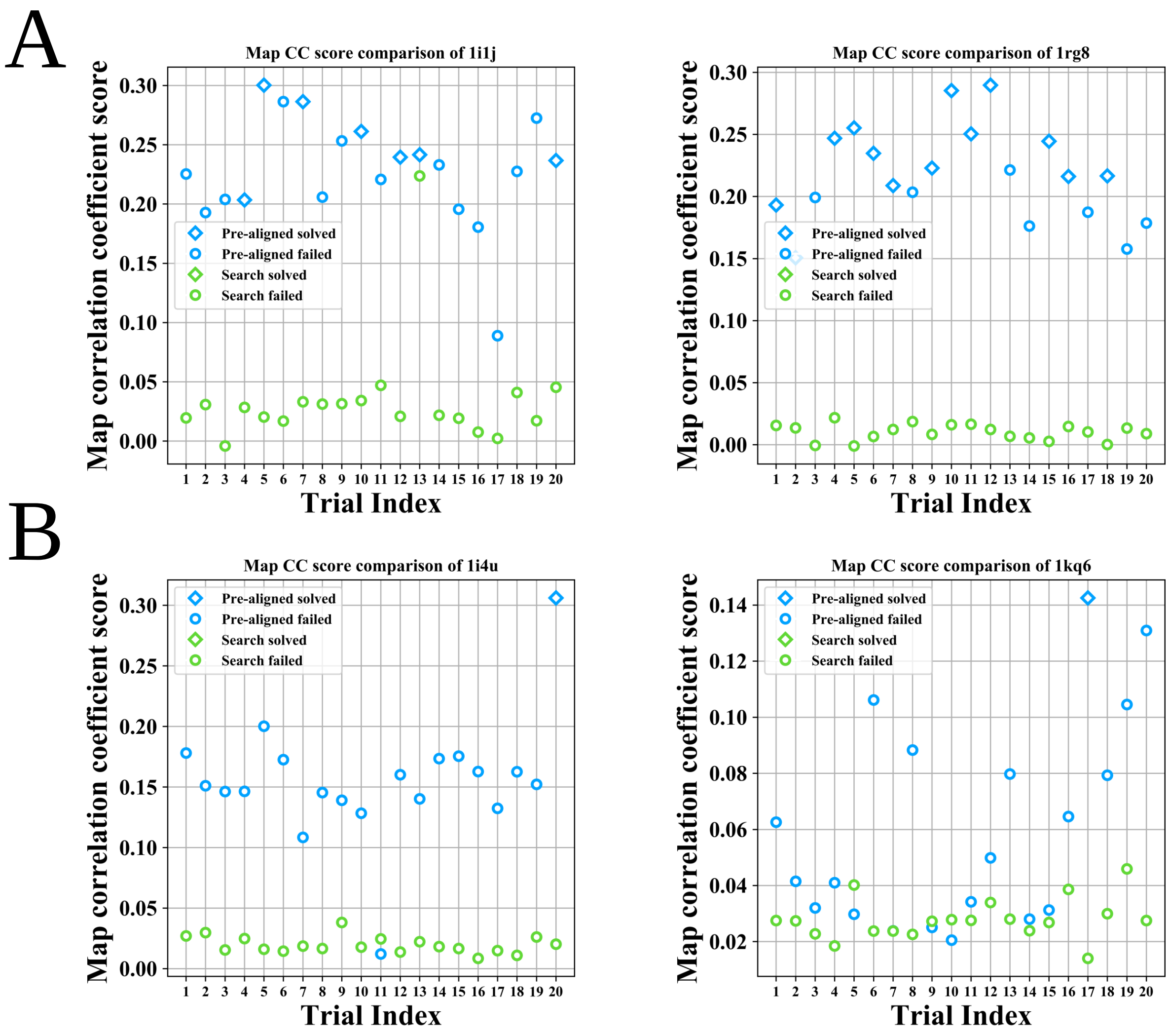

Figure S5: Map-CC for four cases that pre-aligned search of AWSEM-Suite predicted model can solve. For the panel A, most trials of these two cases are successful that indicate the searching procedure is not optimal. For the panel B, only one trial leads to the successful solution but the molecular replacement program are all failed to find.
